# Phylogenetic diversification patterns and divergence times in ground beetles (Coleoptera: Carabidae: Harpalinae)

**DOI:** 10.1186/1471-2148-10-262

**Published:** 2010-08-27

**Authors:** Karen A Ober, Thomas N Heider

**Affiliations:** 1 Department of Biology, College of the Holy Cross, 1 College St., Worcester, MA 01610 USA; 2 Molecular and Cell Biology, University of Connecticut, Storrs, CT 06269 USA

## Abstract

**Background:**

Harpalinae is a species rich clade of carabid beetles with many unusual morphological forms and ecological interactions. How this diversity evolved has been difficult to reconstruct, perhaps because harpalines underwent a rapid burst of diversification early in their evolutionary history. Here we investigate the tempo of evolution in harpalines using molecular divergence dating techniques and explore the rates of lineage accumulation in harpalines and their sister group.

**Results:**

According to molecular divergence date estimates, harpalines originated in the mid Cretaceous but did not diversify extensively until the late Cretaceous or early Paleogene about 32 million years after their origin. In a relatively small window of time, harpalines underwent rapid speciation. Harpalines have a relative high net diversification rate and increased cladogenesis in some regions of the clade. We did not see a significant decrease in diversification rate through time in the MCCR test, but a model of diversification with two shift points to lower diversification rates fit the harpaline lineage accumulation through time the best.

**Conclusions:**

Our results indicate harpalines are significantly more diverse and have higher diversification than their sistergroup. Instead of an immediate burst of explosive diversification, harpalines may have had a long "fuse" before major lineages diversified during the early Paleogene when other taxa such as mammals, birds, and some flowering plants were also rapidly diversifying.

## Background

Ever since Darwin, biologists have been interested in using phylogenetic trees to investigate the pattern and tempo of lineage diversification. We see that the branching patterns in phylogenies contain information about the processes of speciation and extinction. Molecular phylogenies of species rich groups supply hypotheses of evolutionary relationships among higher taxa, and also allow us to test hypotheses about the tempo and pattern of diversification [1-4], revealing both the topology of ancestor-descendant relationships and the tempo of descent among members of a clade. It is clear that organismal diversification rates (speciation minus extinction) have been variable across lineages and through time, with some lineages showing slowing rates of lineage accumulation [5] and others showing rapid diversification [6] or radiations. Radiations are generally defined in a way that includes rapid cladogenesis from a common ancestor [7], yielding taxon rich clades. The tempo and pattern of diversification of many major lineages of organisms, including insects, remain controversial [8] - does macroevolution proceed at a relatively constant rate, accumulating lineages exponentially, or does it proceed through bursts of speciation triggered by processes such as adaptive radiation but otherwise remaining relatively constant?

Rapid or explosive diversifications are characterized by lineages that have diverged in rapid succession within a relatively short time span. Molecular phylogenies can display a pattern that characterizes ancient rapid radiations as its signature [9]. The signature can be described as close temporal spacing of a number cladogenetic or lineage-splitting events in a phylogeny, such that the internal branches (internodes) that link taxa together are very short. Although such a phylogenetic pattern is to be expected from an ancient rapid radiation, the pattern can also be observed in the case of other factors involved in phylogenetic reconstruction such as inadequate data, conflict within or among data sets, loss of phylogenetic signal over time, or data with inappropriate evolutionary rates [10]. Phylogenies of major lineages of insects based on morphological and/or molecular data have sometimes been contentious, often lacking the data to distinguish between alternative evolutionary relationships [11-15]. The difficulty in confidently resolving basal splits in these phylogenies may be a result of insufficient time during cladogenesis to accumulate strong phylogenetic signal in the data because of rapid lineage splitting.

**Figure 1:**
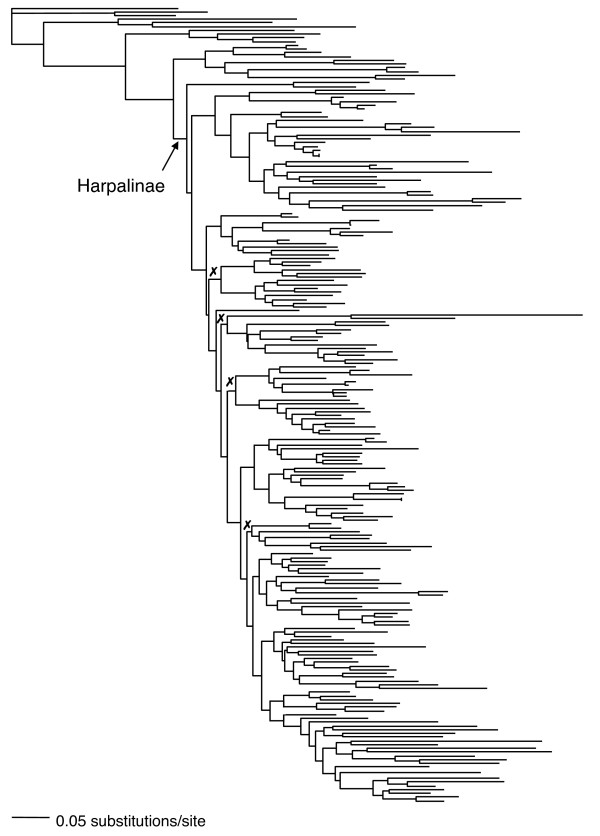
**Maximum likelihood tree and preferred phylogenetic hypothesis of Harpalinae from the 28S+wg molecular data set**. The harpaline clade is indicated with an arrow. Branchlengths were estimated with GTR+Γ+I model of molecular evolution. Redrawn from Ober and Maddison [28] Figure 2. Sistergroup relationships of tribes marked with an X are not present in an alternative topology inferred from combined 18S+28S+wg data in BEAST.

The ground beetle (Carabidae) subfamily Harpalinae may display such a signature of rapid diversification. Harpalinae is the largest subfamily of carabid beetles and includes about 19,811 species [16], the bulk of the family's species-level diversity. Some harpalines exhibit a number of unusual morphological forms such as an elongated body in the genus *Agra*, an ant-like form in *Calybe*, snail-shell cracking mandibles in *Licinus *[17], and an extremely dorso-ventrally flattened body as in *Mormolyce*. Not only are members of Harpalinae diverse in morphological form, but also they display a variety of unusual lifestyles including granivory [18], ovoviviparity [19], symbiosis with ants and termites [20,21], ectoparasitism of other insects [22,23], specialized host mimicry by ectoparasites [24,25], and arboreality [26]. Resolving the tribal relationships within the harpalines has been difficult, in part, because branch lengths at the base of the harpaline clade are very short in molecular based trees (Figure 1; [27,28]) and morphological phylogenetic analyses have not been able to resolve basal relationships within Harpalinae [29]. Evidence from the fossil record that the subfamily Harpalinae underwent an explosive radiation in the Cretaceous period [26,30-32], and the very short branches at the base of the harpaline clade in the molecular phylogeny of three nuclear genes point to the possibility that this speciose group represents a rapid radiation within carabids, and the tribes may have originated almost simultaneously. Harpalines contain more than 30 times the number of species as Brachininae (655 species), their sister group and almost 200 times more than the next closest clade, the austral psydrines (100 species) [16]. Rapid, ancient radiations are a difficult challenge to phylogenetic inference [9,33-35] because short interior branches limit the historical record of early diversification. In Harpalinae, there are also long terminal branches [28] that are prone to the analytical artifact of long-branch attraction [36].

Using the preferred phylogeny of harpalines and close relatives [28], molecular sequence data [27,28], and fossil data, we investigated the timing and tempo of the diversification of harpalines. The aim of this work was to test the hypothesis that harpalines evolved their incredible diversity through an explosive radiation shortly after their evolutionary origin. Our goals were to determine (1) the age of divergence of the major lineages (approximately tribes) of harpalines, (2) whether the evolutionary history of diversification of the subfamily Harpalinae and its brachinine sister group depart significantly from a constant rate model, and (3) whether harpalines and/or brachinines have experienced changes in diversification rates during their evolutionary history indicative of an explosive radiation of lineages. In this study, we report on the timing and patterns of evolution in the largest subfamily of ground beetles and its sister group, the brachinine bombardier beetles.

## Methods

### Phylogenetic tree and molecular sequence data

Using the 28Sbe+wg maximum likelihood tree topology from Ober and Maddison [28] and DNA sequence data sets for 18S from Ober [27] and 28S and *wingless *from Ober and Maddison [28], we estimated branch lengths under the maximum likelihood GTR+Γ+I model in PAUP* [37]. Branch lengths for the all data combined tree were estimated from the 28S data due to the large amount of missing data for many taxa. The 28Sbe+wg likelihood tree [28] represents the best phylogenetic hypothesis available for Harpalinae. It has the most extensive taxon sampling for the subfamily and uses a model of molecular evolution to estimate the phylogeny from two nuclear genes. Monophyly of Harpalinae is based on molecular data [27,28,38,39], morphological data [40], defensive chemical data [41-44], and cytological data [45,46]. Most tribes within the subfamily are monophyletic, but the relationships among tribes are not strongly supported [28]. The effects of phylogenetic uncertainty on divergence dates were not explored in depth in this study. One BEAST analysis was done allowing tree topology to be estimated along with other parameter values. The topology was largely congruent with the 28Sbe+wg maximum likelihood tree topology from Ober and Maddison [28] with a few exceptions noted in Figure 1. A complete list of taxa and DNA sequence data used in this study from Ober [27] and Ober and Maddison [28] is in Additional file 1, Table S1. We estimated divergence dates from combined 18S, 28S and *wg *data and also from each gene separately. For separate gene analyses, taxa for which no molecular data was available were pruned from the tree yielding a 18S tree with 21 harpaline taxa and 14 outgroups, a *wg *tree with 157 harpalines and 16 outgroups, and a 28S tree with 193 harpalines and 18 outgroups. We used a likelihood ratio test to look for evidence of a molecular clock in the molecular phylogenies.

### Divergence time estimation

**Figure 2:**
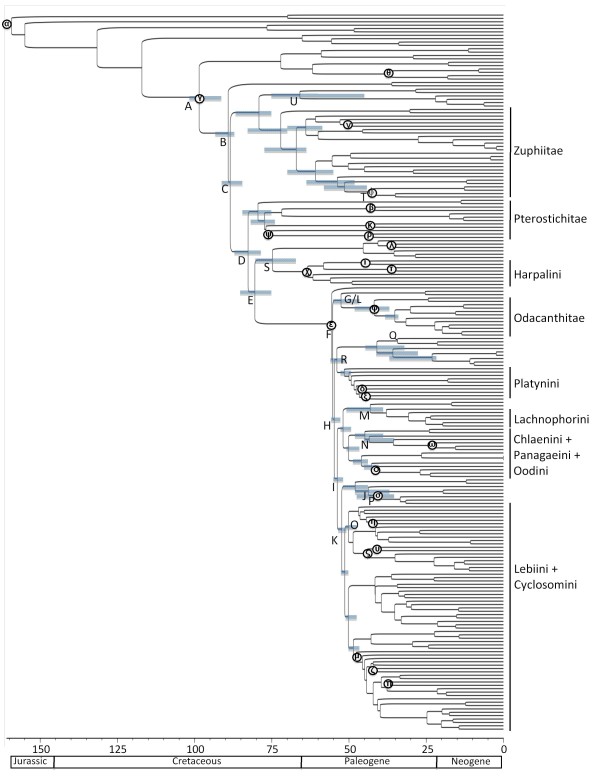
**Harpalinae phylogeny dated using a Bayesian relaxed clock method with all-data-combined in BEAST**. Branches are proportional to time in millions of years. 95% confidence intervals for the ages of basal branches in the tree and major harpaline lineages (tribes) are indicated with blue bars. The capital letters indicate the major lineages of harpalines (see Table 3), and the Greek symbols represent the fossil calibration points used in the molecular dating analyses (see Table 1).

Divergence times were estimated using the penalized likelihood approach provided by the program r8s v.1.71 [47] and a Bayesian relaxed clock method in BEAST v.1.5.4 [48] for all data combined and separate gene datasets. For r8s and BEAST analyses, we calibrated several internal nodes (26 in the all data combined, 28S, and *wg *data sets and 7 in the 18S data set) based on the fossil record (Table 1 and Figure 2).

**Table 1:** Calibration points used for estimation of phylogenetic divergence times for Harpalinae.

	Fossil	Min. age for lineage (Mybp)	Period	Location	Reference
**α**	Carabidae	155 - 160	Late Jurassic	South Kazakhstan and Bavaria	[31,50]
**β**	*Abacetus*	44.1	Middle Eocene	Baltic	[86-88]
**γ**	Harpalinae	89.0 - 93.5	Late Cretaceous	South Kazakhstan	[31]
**δ**	*Agonum*	49.0 - 52.0	Early Eocene	Green River CO	[89]
**ε**	*Amara*	44.1	Middle Eocene	Baltic	[86]
**ζ**	*Cymindoidea*	44.1	Middle Eocene	Baltic	[86]
**η**	*Apristus*	44.1	Middle Eocene	Baltic	[86]
**θ**	*Brachinus*	34.9 - 38.0	Late Eocene	Florissant CO	[89,90]
**ι**	*Bradycellus*	44.1	Middle Eocene	Baltic	[86]
**κ**	*Calathus*	44.1	Middle Eocene	Baltic	[86]
**λ**	*Diplocheila*	34.9 - 38.0	Late Eocene	Florissant CO	[89,90]
**μ**	*Dromius*	44.1	Middle Eocene	Baltic	[86]
**ν**	*Galerita*	49.0 - 52.0	Early Eocene	Green River CO	[89]
**ξ**	*Platynus*	44.1	Middle Eocene	Baltic	[86]
**ο**	*Chlaenius*	44.1	Middle Eocene	Baltic	[86]
**π**	*Plochionus*	34.9 - 38.0	Late Eocene	Florissant CO	[89]
**ρ**	*Pterostichus*	44.1	Middle Eocene	Baltic	[86-88]
**σ**	*Badister*	5.3 - 7.1	Late Miocene	Oeningen	[91]
**τ**	*Stenolophus*	34.9 - 38.0	Late Eocene	Florissant CO	[89,90]
**ν**	*Syntomus*	44.1	Middle Eocene	Baltic	[86]
**ϕ**	Helluonini	44.1	Middle Eocene	Baltic	[86]
**χ**	Harpalini	61.0 - 65.0	Early Paleocene	Staratschin cap	[92]
**ψ**	Pterostichini	80 - 90	Late Cretaceous	Orapa	[93,94]
**ω**	*Panagaeus*	23.8 - 28.5	Late Oligocene	Aix-en-Provance	[95]
**φ**	Odacanthini	34.9 - 38.0	Late Eocene	Florissant CO	[89,90]
**ς**	*Lebia*	44.1	Middle Eocene	Baltic	[86]

Greek symbols correspond to fossil-based minimum age constraints placed on the phylogeny in Figure 2.

The r8s penalized likelihood method with the TN algorithm uses semiparametric rate-smoothing to optimize rate changes among branches. Smoothing parameters were estimated by using the fossil-based model cross-validation feature in r8s [49]. All 26 fossils passed the cross-validation test and were included in the analysis. The outermost outgroup was pruned from the tree. Two r8s analyses were done for each data set, one with the younger fossil dates for each calibration point and one with the older dates. All nodes ages were free to be estimated. All nodes for which there was fossil information were constrained to be no older than a maximum age of 155 (younger dates) or 160 (older dates) My ago, the fossil age of Carabidae [31,50], except the root node of the tree which was constrained with a maximum age of 197 My ago, the age of Adephaga [50], with no minimum age constraint. Minimum age constraints were applied to nodes according to fossil information (Table 1).

We also inferred divergence dates using a Bayesian relaxed clock uncorrelated lognormal method in BEAST for all data combined and the three genes separately. For the all data combined analysis, we concatenated the three data sets for all taxa, but created three data partitions for 18S, 28S, and *wg*. We chose a separate GTR+I+Γ model with four gamma categories, based on MrModelTest [51]; a Yule process of speciation, and an uncorrelated lognormal relaxed clock model of rate variation for each partition. Model parameters were unlinked across partitions. For the separate gene analyses, we applied a GTR+I+Γ model with four gamma categories, a Yule process of speciation, and an uncorrelated lognormal relaxed clock model of rate. The tree topology prior was fixed for all BEAST analyses except one all-data-combined analysis where the tree topology was estimated along with other parameter values. The same calibration points as in the r8s analyses were used (Table 1). Node constraints were assigned a normal prior distribution with means equal to the midpoint of the fossil date and the standard deviations encompassing the youngest and oldest age of each fossil. A normal distribution was chosen because it allows uncertainty in the calibration estimates [52]. After an initial period of fine-tuning the operators, two separate MCMC analyses were run for 70 million generations for each data set with parameters sampled every 1000 generations. Independent runs were combined using LogCombiner1.5.4 [53], and the first 20% of the generations from each run was discarded as burnin. Convergence of the chains was checked using TRACER 1.4 [54]. The searches achieved adequate mixing as assessed by the high effective sampling size (ESS) values for all parameters of 100 or greater. Node ages and upper and lower bounds of the 95% highest posterior density interval for divergence times was calculated using TreeAnnotator 1.5.4 and visualized using FigTree 1.3.1 [55].

### Biodiversity estimate

To test if the Harpalinae clade (19,811 species) is more diverse than its sister clade, the Brachininae (655 species), we determined if the two groups had significantly different number of extant species using a conservative test of Slowinsky and Guyer [56].

### Diversification rates

Several diversification statistics were run on the chronograms for each clade (Harpalinae and Brachininae) and for the overall tree. All diversification tests were performed in R using APE [57], GEIGER [58], and LASER [59].

The overall rates of net diversification for the Harpalinae clade and the Brachininae clade were estimated from the pruned all-data-combined BEAST and r8s chronograms using GEIGER based on a pure-birth process of diversification and also with a speciation:extinction rate of 2:1 using the method of Magallón and Sanderson [60]. The rate estimate took into account the extant taxa missing from each clade not sampled in the chronograms.

The relative cladogenesis (RC) statistic, which uses a broken-stick distribution to identify branches in the tree ancestral to a greater proportion of extant descendant lineages than expected by chance [1,58], was used to calculate the probability that a particular lineage at time *t *will have *k *descendants given the total number of descendants at time 0 (the present). This test detects and locates unusually rapid shifts in diversification rates by looking for unusually lineage-rich clades in the cohort of clades all originating at one slice of time in the tree. We estimated the RC statistic with Bonferroni correction as implemented in GEIGER for the all-data-combined r8s and BEAST chronograms.

Rates of cladogenesis through time were investigated in the Harpalinae and Brachininae all data combined BEAST chronograms using constant rate (CR) test of Pybus and Harvey [3] that estimates the gamma statistic (γ) of a given chronogram. Under a Yule (pure birth) process, γ values of completely sampled phylogenies have been shown to fit a standard normal distribution with mean = 0 [3,61]. Significantly negative values of γ (γ < -1.645 for a one-tailed test) are indicative of decreasing rates of cladogenesis through time (i.e. internal nodes are distributed more toward the root than expected under a pure birth process). However, incomplete taxon sampling has been shown to inflate the type I error of the CR test [3]. To correct for the under sampling in our analysis (193 of 19,811 extant harpalines and 10 of 655 extant brachinines), we used Monte Carlo constant rate (MCCR) test [3] in which full topologies for each clade were simulated under the Yule process and then randomly sub sampled in Mesquite [62] to generate a corrected null distribution. We compared our observed γ for brachinines and harpalines to the appropriate null distribution based on 1000 simulated trees. We calculated γ using the LASER package in R for each of these simulated trees and then used the resulting set of values as our null distribution under incomplete taxon sampling [57]. For both the MCCR tests, we performed a one-tailed test with a critical value of *p *= 0.05.

Rates of diversification were evaluated using Lineage Through Time (LTT) plots, which illustrate the accumulation of lineages over time for the all data combined BEAST chronograms for the ingroup Harpalinae and its sister group Brachininae using APE. To evaluate the effects of incomplete taxon sampling on the slope of the LTT plot we used 1000 subsampled simulated Yule process brachinine and harpaline trees to construct mean LTT curves for comparison with the empirical LTT curves.

We evaluate the fit of six models of diversification [63,64] in LASER using Akaike Information Criteria (AIC) and/or hierarchical likelihood ratio tests (hLRTs) for the full all-data-combined BEAST chronogram, the harpaline clade and the brachinine clade. Model 1 (Pure Birth) assumes a pure birth or Yule model of diversification, Model 2 (Birth Death) assumes a constant birth death model of diversification, Model 3 (SPVAR) specifies a variable speciation rate and constant extinction rate, Model 4 (EXVAR) specifies a variable extinction rate and constant speciation rate, Model 5 (yule2rate) assumes an abrupt change in diversification rate at some breakpoint in the past, Model 6 (yule3rate) assumes three different diversification rates with two breakpoints in the past. For Models 5 and 6 we evaluated the hypothesized shifts in diversification rate every one million years.

## Results

### Divergence time estimations of Harpalinae

All three molecular datasets rejected a strict molecular clock for the tree (18S χ^2 ^= 305.49, d.f. = 33, p < 0.001; *wg *χ^2 ^= 526.47, d.f. = 171, p < 0.001; 28S χ^2 ^= 2493.55, d.f. = 211, p < 0.001).

Phylogenetic relationships of tribes within harpalines estimated by BEAST from all data combined were largely congruent with the 28Sbe+wg maximum likelihood tree topology from Ober and Maddison [28]. For the most part, tribes and supertribes were monophyletic except for Perigonini. However, some of the sistergroup relationships of tribes differed between the Ober and Maddison [28] and BEAST tree (Figure 1). The difference in tree topology did not affect the estimate of the age of the origin of Harpalinae (102.1 My ago), but the origin of most of the tribes was estimated to be slightly older than in the Ober and Maddison [28] tree. Nonetheless, by the Eocene, 58 My after the origin of the subfamily, all tribes are present, similar to the timing of diversification estimated from the Ober and Maddison [28] tree.

**Figure 3:**
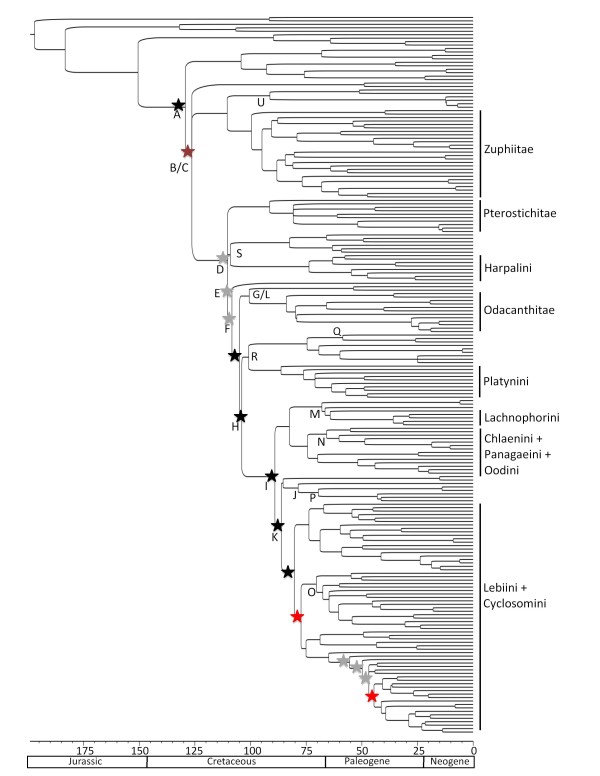
**Results of the relative cladogenesis (RC) test for the Harpaline chronogram generated with r8s with all-data-combined**. Branches are proportional to time in millions of years. The capital letters indicate the major lineages of harpalines (see Table 2). The stars mark branches where there was a significant increase in diversification rate (with Bonferroni correction). The black stars are nodes where the increase in diversification rate was detected in both the BEAST chronogram and r8s chronogram. The gray stars are nodes that showed an increase in diversifciation rate only in the r8s chronogram. The red stars show a more conservative interpretation of branches that have experienced increased diversification and are not subject to the effects of unusually diverse daughter clades. The dark red star is one such of these clades in the BEAST chronogram.

Our estimate for the origin of Harpalinae ranged from about 92 My ago to about 153 My ago (Figures 2 and 3, Tables 2 and 3) depending on the type of analysis and the gene(s) used to estimate the divergence times. r8s estimates of divergence times were generally older than the BEAST estimates, outside the 95% confidence intervals for the BEAST estimates. r8s divergence date estimates for the origin of harpalines from all DNA data sets were much older (~120 My ago) than to the fossil record (~91 My ago). Results of divergence date estimates from individual genes showed that the oldest dates for all nodes were inferred from the *wg *gene and the youngest dates for all nodes were inferred from the 18S gene with r8s (Table 2). The results of the BEAST analyses did not show a clear bias of particular genes for older or younger date estimates (Table 3).

**Table 2:** Divergence dates of harpaline lineages estimated using r8s.

		Date of origin (My bp)			
	**Lineage**	**Combined**	**18S**	***wg***	**28S**

**A**	Harpalinae	129.3-133.4	120.0-120.4	153.1	124.7-129.0
**B**	Morionini	126.4-130.6	95.3-95.6	139.5	122.1-126.6
**C**	Zuphiitae+Ctenodactylini	126.4-130.6	90.8-91.1	132.5	122.1-126.6
**D**	Pterostichites	110.3-115.6	67.5-67.8	132.5	107.3-112.9
**E**	Harpalini+Licinini	110.3-114.3	-	130.8	107.3-112.9
**F**	*Amara*	108.4-113.5	65.0-65.3	119.8	105.5-110.9
**G**	Odacanthitae	100.6-105.3	-	117.8	98.9-103.9
**H**	Platynini+Pseudomorphini *et al*.	104.0-108.8	51.7-57.5	113.9	101.7115.0
**I**	Oodini+Lachnophorini	89.1-92.5	57.2-57.5	113.2	87.5-91.0
**J**	*Badiste*r+Perigonini	78.6-81.5	-	99.6	78.2-81.2
**K**	Lebiini	86.2-89.4	57.2-57.5	106.4	84.8-88.1
**L**	Peleciini	100.6-105.3	64.4-64.7	117.8	98.9-103.9
**M**	Lachnophorini	68.0-70.4	57.2-57.5	98.0	67.1-69.6
**N**	Panagaeini	60.2-62.1	-	82.5	59.1-61.1
**O**	Cyclosomini	73.8-76.1	43.5-43.8	96.3	72.9-75.3
**P**	Perigonini	69.6-72.1	-	95.3	69.8-72.5
**Q**	Graphipterini	58.6-61.3	-	72.7	59.5-62.4
**R**	Platynini	100.8-105.6	51.7-52.0	103.5	98.5-130.4
**S**	Harpalini	109.1-114.3	67.5-67.8	111.0	105.8-111.3
**T**	Helluomorphini	60.5-63.0	-	83.9	60.6-63.2
**U**	Ctenodactylini	83.9-94.8	-	101.5	88.0-91.5

Estimated age of stem lineage origins within Harpalinae from all-data-combined and18S rDNA, *wingless*, and 28S rDNA data sets from r8s relaxed clock analyses using the youngest hypothesized age of the fossil calibration (first number) and the oldest hypothesized age of the fossil calibration (second number) from Table 2. Young and old ages resulted in essentially identical estimates for the *wg *gene. Not all nodes were present in the 18S data set due to limited taxon sampling [27]. The capital letters correspond to clades in Figure 3.

**Table 3:** Divergence dates estimated using BEAST.

Date of origin (My bp)									
		Combined		18S		*wg*		28S	
	Lineage		95% CI		95% CI		95% CI		95% CI
**A**	Harpalinae	98.5	92.1-102.4	105.2	94.3-118.9	100.3	91.5-112.9	92.3	89.0-95.7
**B**	Morionini	89.1	88.4-93.5	91.6	89.0-94.2	91.5	88.9-94.0	90.3	87.9-92.7
**C**	Zuphitae+Ctenodactylini	88.5	84.8-91.7	90.1	85.2-93.6	81.9	83.8-92.0	89.0	85.9-91.8
**D**	Pterostichites	82.7	79.8-87.7	85.3	80.4-90.3	87.3	79.4-89.2	76.9	72.1-81.9
**E**	Harpalini+Licinini	80.5	75.8-86.2	-	-	81.4	70.3-90.4	65.7	61.7-69.9
**F**	*Amara*	55.6	54.0-56.5	48.4	47.1-49.8	50.1	48.9-51.6	55.6	54.3-56.8
**G**	Odacanthitae	52.6	47.9-55.8	-	-	46.3	40.3-50.4	54.6	52.6-56.4
**H**	Platynini+Pseudomorphini *et al*.	54.8	53.4-56.0	47.9	46.5-49.2	49.7	48.4-51.1	55.3	54.1-56.6
**I**	Oodini+Lachnophorini	53.8	52.6-55.2	40.4	27.5-47.2	49.5	48.2-50.9	54.0	52.6-55.4
**J**	*Badiste*r+Perigonini	43.7	37.8-49.0	-	-	37.3	22.4-48.7	47.3	39.4-53.7
**K**	Lebiini	52.3	51.4-54.3	40.4	27.5-48.7	48.8	47.4-50.2	53.1	51.6-54.6
**L**	Peleciini	52.6	47.9-55.8	48.1	46.7-49.4	46.3	40.3-50.4	54.6	52.6-56.4
**M**	Lachnophorini	43.1	39.9-51.1	34.2	18.6-47.2	41.6	31.7-48.9	43.0	38.3-47.8
**N**	Panagaeini	43.5	35.5-46.3	-	-	40.0	33.4-45.9	39.5	35.2-44.1
**O**	Cyclosomini	50.2	48.5-52.1	27.3	11.7-42.1	47.4	45.8-49.0	50.8	48.8-52.7
**P**	Perigonini	41.8	36.0-48.3	-	-	24.4	7.7-40.8	33.6	24.8-42.8
**Q**	Graphipterini	34.3	24.6-41.1	-	-	25.8	13.8-37.6	36.7	31.3-42.3
**R**	Platynini	53.9	52.3-55.0	47.4	46.1-48.7	44.1	37.5-49.6	54.5	52.9-56.1
**S**	Harpalini	74.8	68.2-81.1	80.5	70.1-88.5	67.7	56.5-79.7	64.8	61.1-68.7
**T**	Helluomorphini	42.7	42.2-46.1	-	-	44.0	42.1-45.9	45.5	43.6-47.3
**U**	Ctenodactylini	65.9	44.5-75.5	-	-	58.5	36.9-80.0	60.1	51.1-72.0

Estimated age of stem lineage origins within Harpalinae from the all-data-combined and separate 18S rDNA, *wingless*, and 28S rDNA data sets from BEAST analyses. Not all nodes were present in the 18S data set due to limited taxon sampling [27]. The capital letters correspond to clades in Figure 2.

The combined molecular data suggest harpalines originated approximately 115 MY ago (Tables 2 and 3), but may not have diversified much until ~30 My after the origin of the subfamily. Our analyses suggest that many harpaline tribes probably originated ~80 My ago with most of the extant genera evolving 25 - 40 My ago. The average age estimates of most of the tribes imply that they originated within a 38 My time window (~47 - 85 My ago) about 30 My after the origin of Harpalinae (Tables 2 and 3, Figures 2 and 3). Approximately 68 My elapsed from the origin of harpalines to the time when all major lineages and tribes are present.

### Tempo of diversification

Although the sister groups are the same age and descended from a common speciation event, there is a significant difference (p = 0.03) in species number between Harpalinae and Brachininae, indicating an increase in net diversification (speciation minus extinction) within the harpaline clade. The clade that contains harpalines is also significantly more diverse (p = 0.008) than the sister group to brachinines + harpalines, the austral psydrines, which contains only 100 extant species (Table 4).

**Table 4:** Comparison of species richness between clades.

Subfamily	**# of species **[14]	
Harpalinae	19,811	*p *= 0.032
Brachininae	655	
Austral psydrines	100	

A conservative Slowinsk and Guyer test, to determine if clades differ in number of species, shows Harpalinae with significantly more species than Brachininae (p = 0.032 [56]).

The estimated overall net diversification rate with a pure birth model of diversification in brachinines was 0.080 per My (0.076 per My with 2:1 speciation:extinction rate) with the BEAST all-data-combined chronogram to 0.056 per My with the r8s all data combined chronogram, and in harpalines the estimated overall net diversification rate with a pure birth model was 0.103 per My (0.10 per My with 2:1 speciation:extinction rate) with the BEAST chronogram to 0.073 per My with the r8s chronogram.

The RC test indicated a significant difference in cladogenesis rate between harpalines and brachinines. We located unusually rapid shifts in diversification in the node subtending Harpalinae and along at least four basal branches of harpalines (~80 - 120 My ago in the r8s chronogram, Figure 3, and ~50 - 65 My ago in the BEAST chronogram). There were also significant increases in diversification within and the tribe Lebiini, especially the subtribe Calleidina (Figure 3).

Results of the CR and MCCR tests showed the observed value of γ in the Harpalinae clade was negative (γ = -10.606), which rejects the hypothesis that rates of lineage accumulation remained constant over time, however, when compared to the distribution of γ statistics of the simulations, the harpaline γ statistic was not statistically significant (MCCR test: *p *= 0.98). The brachinine γ statistic was not significantly different than zero, and the CR and MCCR tests do not reject a constant rate of diversification (γ = 0.611, CR test: *p *= 0.27; MCCR test: *p *= 1.0).

**Figure 4:**
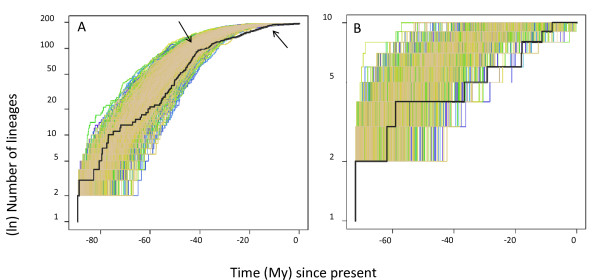
**Semi-logarithmic plot of Lineages Through Time for the Harpalinae clade only (A) and the Brachininae clade only (B)**. The dark black lines are the empirical chronograms from the BEAST analysis of all data combined. The lighter colored lines are simulations with incomplete taxon sampling generated under a pure birth process. Upturns and down turns in the empirical LTT plots reflect changes in rates of diversification. The arrows in the Harpaline LTT (A) show changes in diversification rates are estimated to have occurred according to the best fitting model of lineage diversification.

The semilogarithmic LTT plots for the harpaline and brachinine clades are shown in Figure 4. The accumulation of harpaline lineages did not differ significantly from a constant rate of diversification early in harpaline evolution when compared to 1000 simulated Yule process trees (light lines in Figure 4). However, there was a steep increase in the number of harpaline lineages early after the origin of the subfamily, approximately at 75 My ago, and a shift in rate at about 41 My ago (arrow in Figure 4). This was the time period when most harpaline tribes were diversifying according to the BEAST divergence time estimates. Brachinines showed a fairly slow but constant rate of lineage accumulation (Figure 4), within or slightly below the range of simulated trees.

For the harpaline clade, a model of two abrupt rate shifts, Model 6: yule3rate (AIC = -2.60), was chosen over other constant and variable rate models (Table 5). A diversification rate decrease (from 0.08 to 0.02) was estimated to have happened first at 41.34 My ago and then again at 11.87 My ago (from 0.02 to 0.004) (arrows in Figure 4). Statistical analyses of the LTT plot for brachinines indicated that a Pure Birth model of constant rate of diversification (Model 1, AIC = 54.42) could not be rejected (Table 5). A model of two abrupt rate shifts, Model 6: yule3rate (AIC = 32.88) best fit the overall tree with decreases in diversification rates occurring at 40.04 My ago and 11.07 My ago (Table 5).

**Table 5:** Testing models of diversification in Harpalinae and Brachininae.

Clade	Pure Birth lnL (AIC)	Birth Death lnL (AIC)	SPVAR lnL (AIC)	EXVAR lnL (AIC)	yule2rate lnL (AIC)	yule3rate lnL (AIC)	γ statistic
Harpalinae	-65.18 (132.36)	-65.18 (134.36)	-14.73 (35.46)	-66.43 (138.87)	-12.73 (31.45)^a^	**6.30 (-2.60)^b^**	-10.606
Brachininae	**-26.21 (54.42)**	-26.21 (56.42)	-26.10 (58.19)	-26.23 (58.46)	--25.06 (56.12)^c^	-23.28 (56.55)^d^	0.611
Entire Tree	-67.78 (139.56)	-67.78 (139.56)	-50.98 (107.97)	-69.06 (144.13)	-26.44 (58.87)^e^	**-11.44 (32.88)^f^**	

Pure Birth assumes a pure birth or Yule model of diversification, Birth Death assumes a constant birth death model of diversification, SPVAR specifies a variable speciation rate and constant extinction rate, EXVAR specifies a variable extinction rate and constant speciation rate, yule2rate assumes an abrupt change in diversification rate at some breakpoint in the past, yule3rate assumes three different diversification rates with two breakpoints in the past.

^a ^breakpoint at 40.25 My ago

^b ^breakpoints at 11.78 and 41.34 My ago

^c ^breakpoint at 59.01 My ago

^d ^breakpoints at 17.22 and 18.76 My ago

^e ^breakpoint at 33.39 My ago

^f ^breakpoints at 11.07 and 40.05 My ago

## Discussion

### Divergence time estimations of Harpalinae

We found a mid-Cretaceous origin of Harpalinae with most tribes arising by the Paleocene. Crown diversification of tribes appears to have commenced by the late-middle Eocene, and nearly all tribes appeared by the late Eocene. In a relatively short amount of time (about 35 My), harpalines diversified from just a few lineages into almost all the major lineages extant today. From the divergence date estimates, it appears this rapid diversification was not immediately after the origin of harpalines, but about 32 My later.

BEAST estimates of divergence dates from all molecular data combined were slightly older than the fossil record. r8s estimated divergence dates much older that the fossil record (about 40 My) and the BEAST estimates (about 30 My). Differences between the r8s and BEAST estimates may due to the relaxed clock method used to infer dates and the prior distributions for the divergence time of calibration points in BEAST versus the minimum and maximum age constraints in r8s. We prefer the dates from the all data combined BEAST analyses as a more accurate estimate of harpaline evolution based on the partitioned model of molecular evolution and Bayesian framework. Estimates of divergence dates from molecular data are subject to several sources of error. Estimates may be subject to errors in fossil calibration points or topology, inappropriate priors, or invalid clock assumptions, including much more rate heterogeneity than can be accommodated by the programs we used. The error introduced by finite sequence data or model misspecification, such as neglect of multiple hits and across site rate variation [65] can cause spurious results in divergence time estimates. Sources of error have been reviewed elsewhere [[66-68], among others]. We did not explicitly explore the effect of differences in tree topology on divergence time estimates. We assumed the Ober and Maddison [28] 28S+wg maximum likelihood tree was the best phylogenetic hypothesis for harpalines available, but results from the BEAST analysis of all data combined where topology was also estimated, did not change our conclusions about the timing of harpaline origin or the rapidness of diversification of tribes. Our estimates of harpaline divergence dates are a working hypothesis subject to the addition of more taxa, molecular data, improved phylogenetic hypotheses, and fossils yet to be discovered.

### Tempo of diversification

Harpalines have had an increase in net diversification compared to their sister group, the brachinines. It is difficult to tease apart whether this is due to increased speciation or reduced extinction, because the net diversification rate is simply the difference between speciation and extinction rates. We tested for a change in extinction rate through time in harpalines and brachinines, and a change in extinction rate does not explain the difference in species number between the sister groups because he estimated extinction rate for both is the same (0.001). The net diversification rate for harpalines (0.103) is higher than the rate estimated for explosive diversification of angiosperms (0.077 per My) [60] and on par with the rapid mammalian radiation (0.15) [69].

An increase in diversification is evident in the RC test where several harpaline clades have experienced higher than usual cladogenesis. The time of increased cladogenesis occurs approximately 50 - 65 My ago, during a time of incredible faunal and floral turnover in many plant and animal groups. In contrast, there were not shifts in diversification in brachinines or austral psydrines. The RC test is sensitive to temporal depth, phylogenetic scope, and nonindependence of diversification rate shifts [70]. Therefore rate shifts identified by RC should be interpreted with caution, especially nested shifts. If a clade is especially diverse, then one or more of its parent clades will also show significant diversification through a "trickle-down" effect [58]. This may be what is being observed along the basal braches of Harpalinae, and a conservative interpretation of the RC results would be to attribute the increase in diversification rate to the shallowest significant branch (marked with red stars in Figure 4). In this case, there are significant increases in diversification at the base of the harpaline subfamily and within the tribe Lebiini, the largest tribe of Harpalines with over 4,200 species. Alternatively, a more complex series of radiations may have taken place sequentially. An increase in diversification could have taken place over a longer period of time and shifts in diversification could have occurred over several nodes or branches.

Evidence of shifts in diversification rates in harpalines is harder to interpret from the CR and MCCR tests and the LTT plots. Older lineages have a higher risk of being extinct, and thus not sampled, than younger ones (a bias towards more nodes closer to the tips of the tree). Incomplete taxon sampling can give the appearance of nodes near the root of the tree giving rise to more extant descendants than nodes near the tips and are therefore more likely to be included in a small random sample (a bias toward more nodes closer to the root of the tree) [71,72]. The MCCR test for harpalines did not indicate that nodes were closer to the root than expected under a constant rates model after correcting for incomplete taxon sampling. The taxa included in our phylogenetic tree did not represent a random subsample of the total harpaline species, as assumed by the MCCR test with Yule process simulations. Instead, they most likely represent an oversampling of deeper lineages and an undersampling of closely related lineages, which may have affected the conclusions drawn from this test. Furthermore the MCCR test suffers from low statistical power [3] and has been shown to have decreased sensitivity in detecting an initial high rate of diversification followed by a shift to a lower rate [73].

The harpaline LTT plot with simulated constant rate trees agreed with the results of the MCCR test in that it did not show significantly different rate shifts from trees simulated under a Yule process, but the harpaline LTT plot showed a decrease in diversification rate after about 41 My ago, the period after the origin of tribes. Although the simulated trees diversified at a constant rate (resulting in a straight line in a semi-log plot), randomly removing taxa generated an apparent deceleration of lineage accumulation (Figure 4). As with the MCCR test, our tree is not a random subsample of harpaline taxa. This bias may affect how the LTT plot is interpreted. The LTT plot and associated confidence interval generated by simulations is just one method by which timing and tempo of diversification can be visualized and evaluated, other lines of evidence suggest a fairly rapid accumulation of harpaline lineages in the Paleocene and Eocene.

The shift in diversification rate from a high rate initially to lower rates later in harpaline evolutionary history are evident in the results of the tests of models of diversification (Table 5). An increase in extinction rate later in harpaline evolutionary history does not explain the pattern of high diversification early and declining diversification through time. The pattern of lineage accumulation through time rejected a model of changing extinction rate. The best fitting model for harpalines shows a decrease in rate at 41 My ago and again at about 12 My ago. The first rate decrease corresponds well to the end of tribe diversification estimated in BEAST, and perhaps the second shift, which is much more subtle signals the end of a burst of generic diversification, but more taxa will need to be included to test this hypothesis.

Estimated dates of divergence, the RC test, evidence from the LTT plot, and models of diversification tests, seem to suggest a late Cretaceous through middle Eocene period of rapid diversification in harpalines. The Paleogene was a time of profound reorganization of biota, perhaps in part due to a period of warm "greenhouse" climate during the Paleocene/Eocene thermal maximum or continental fission during the late Cretaceous. The late Cretaceous to middle Eocene is the same time period when many other plant and animal taxa are experiencing tremendous changes in evolution from mass extinctions at the K-T boundary to explosive radiations in birds [74], mammals [69] and some plants [75] and insects [6,50,76-78]. Harpaline diversification occurred just after the angiosperm Cretaceous radiation 130 - 90 My ago, when flowering plants achieved widespread floristic dominance for the first time [79,80]. It does not appear that harpalines necessarily co-evolved with angiosperms, but the angiosperm radiation, along with other biotic and abiotic factors, during this period provided harpalines with the possibility of novel ecological interactions. Although the vast majority of harpalines are predatory, the evolving complex angiosperm habitats may have afforded harpalines with new niches in which to diversify, especially because some major groups of harpalines, like the species rich tribe Lebiini, evolved arboreal lifestyles instead of ground dwelling [81]. Recent studies have proposed that another species rich predatory insect group, ants, may have also radiated primarily in the late Cretaceous [82] or Paleogene [83].

## Conclusions

Harpaline species diversity is remarkable compared to its relatives and other carabid clades. Their high rates of net diversification have made them an evolutionary success story and an important part of many modern ecosystems. From the dates of divergence estimates, there was a large amount of rapid speciation in a short amount of time in the late Cretaceous and early Paleogene, but it does not appear that it was a particularly explosive radiation immediately after their evolutionary origin. Instead, harpalines underwent a somewhat elevated bursts of diversification about 30 My after their origin. This suggests that harpalines may have had a long "phylogenetic fuse" [84] before tribes and major lineages began to diversify as seen in birds [74] and mammals [69].

Relationships of tribes and within the carabid subfamily Harpalinae are not strongly supported [27,28]. The lack of support from the data for deep branches in the harpaline molecular phylogeny could be explained by a rapid radiation or cladogenesis during the evolutionary history of this group. Short internal branches created by rapid cladogenesis leave a meager record of diversification that is further potentially obscured by long terminal branches. This explanation was supported in a study attempting to resolve early metazoan evolution [9] and supported by Fiala and Sokal's [85] simulations. They suggested that short internal branches and long terminal branches reduces the accuracy with which a phylogeny can be estimated, hence the resolution that can be obtained.

The pattern seen in the molecular phylogenetic trees of short internal nodes at the base of harpalines with longer terminal branches [27,28] can be explained in several ways. It may be the case that harpalines did undergo a burst of diversification during the late Cretaceous and early Paleogene, but the taxon sampling in the molecular phylogeny was not complete enough to distinguish the pattern clearly. Incomplete taxon sampling in such a large group makes it difficult to interpret the results of the constant rate tests and LTT plots as a significant burst of diversification in Harpalinae evolutionary history. It may be the case that harpalines did diversify rapidly, but it was not as explosive as carabidologists have assumed. On the other hand, the tempo of harpaline evolution may have been fairly constant though time with a high (compared to brachinines), but steady rate of diversification and lineage accumulation, leading to the large and speciose clade we see today. Alternatively, the molecular phylogenies inferred from the three nuclear genes with short basal branches may be due to the information content and phylogenetic utility of the genes or systematic errors in selecting models of evolution for phylogenetic inference. In any case, the inclusion of additional harpaline and brachinine taxa and new molecular data would help shed light on the evolutionary history of the Harpalinae and the patterns and tempo of diversification of this group of beetles.

## Authors' contributions

KAO conceived of the study, carried out some of the analyses, and drafted the manuscript. TNH participated in the design of the study and performed many of the analyses. Both authors read and approved the final manuscript.

## Supplementary Material

Additional file 1 **Table S1**. Taxa and GenBank numbers used in this study. Click here for file
