# Supplementary material for: Phylogenetic diversification patterns and divergence times in ground beetles (Coleoptera: Carabidae: Harpalinae)
Source: BMC Evol Biol. 2010 Aug 27;10:262. doi: 10.1186/1471-2148-10-262 (PMC2939577; doi:10.1186/1471-2148-10-262)
Supplement: Additional file 1 — Table S1. Taxa and GenBank numbers used in this study. [file 1471-2148-10-262-S1.DOC]

Additional File 1

**Table S1. Taxa and GenBank numbers used in this study**

| **Tribe** |  | **28S GenBank** | **wingless GenBank** | **18S GenBank** |
| --- | --- | --- | --- | --- |
| **Subtribe** | **Species** | **Number** | **Number** | **Number** |
| Patrobini | Diplous californicus Motschulsky | AF398699 | AF398587 | AF002785 |
|  | Patrobus longicornis Say | AF398700 | AF398613 | AF002786 |
| Bembidiini | Bembidion levettei carrianum Casey | AF389647 | AF398571 | AF002792 |
| Psydrini | Psydrus piceus LeConte | AF398684 |  | AF002784 |
|  | Laccocenus ambiguus Sloane | AF398675 | AF398596 | AF012486 |
|  | Nomius pygmaeus Dejean | AF438100 | AF437971 |  |
| Austral psydrines | Amblytelus curtus Fabricius | AF398683 | AF398566 | AF012484 |
|  | Melisodera picipennis Westwood | AF398640 | AF398602 | AF012481 |
|  | Meonis sp. | AF398692 | AF398603 | AF398722 |
|  | Mecyclothorax vulcans Blackburn | AF398648 | AF398601 | AF012482 |
| **BRACHININAE** |  |  |  |  |
| Crepidigastrini | Crepidogaster atrata Peringeuy | AF438046 |  |  |
|  | Crepidogaster (Tyronia) ambreana Deuve & Mateu | AF438047 |  |  |
| Brachinini | Brachinus (Neobrachinus) hirsutus Bates | AF398693 | AF398572 | AF012478 |
|  | Brachinus (Metabrachinus) armiger Dejean | AF398682 |  | AF012479 |
|  | Pheropsophus aequinoctialis Linné | AF398678 | AF398619 | AF012477 |
|  | Pheropsophus sp. | AF398658 | AF398618 |  |
|  | Aptinus displosor Dufour | AF398638 | AF398569 | AF012480 |
|  | Aptinus alpinus Dejean & Boisduval | AF398710 | AF398568 |  |
|  | Styphlodromus sp. | AF398641 | AF398628 | AF398718 |
|  | Styphlomerus vittaticollis Péringuey | AF438139 | AF438001 |  |
| **HARPALINAE** |  |  |  |  |
| Metiini | Metius sp. | AF398654 | AF398604 | AF012475 |
|  | Abropus carnifex Fabricius | AF438008 | AF437897 |  |
| Loxandrini | Loxandrus n. sp. nr.amplithorax Straneo | AF398661 | AF398600 | AF002778 |
|  | Oxycrepis (Stolonis) n. sp. | AF438111 | AF437977 |  |
|  | Adrimus n. sp. | AF438011 | AF437899 |  |
| Pterostichini | Pterostichus melanarius Illiger | AF398707 | AF398623 | AF002779 |
|  | Poecilus scitulus LeConte | AF398677 | AF398620 |  |
| Zabrini | Amara apricaria Paykull |  | AF398565 | AF002774 |
|  | Amara (Curtonotus) sp. | AF398694 |  |  |
| Morionini | Morion aridus Allen | AF398698 | AF398606 | AF398721 |
|  | Moriosomus seticollis MacLeay | AF398701 | AF398607 |  |
| Abacetini | Abacetus sp. | AF398681 | AF398635 |  |
|  | Abacetine | AF438007 | AF437896 |  |
| Caelostomini | Caelostomus (Catalainus) n. sp. | AF398704 | AF398573 |  |
| Cnemalobini | Cnemalobus sulciferus Philippi | AF398706 | AF398580 | AF012474 |
| Peleciini | Pelecium n. sp. nr.sulcipenne Chaudoir | AF398672 | AF398614 | AF398715 |
|  | Eripus nitidus Chaudoir | AF398642 | AF398589 | AF398716 |
| Catapieseini | Catapiesis brasiliensis Gray | AF398645 | AF398577 | AF012476 |
| Platynini | Glyptolenus sulcipennis Chaudoir | AF398671 | AF398592 |  |
|  | Agonum extensicolle Say | AF398643 | AF398564 | AF002775 |
|  | Synuchus dubius LeConte | AF398674 | AF398629 |  |
|  | Calathus ruficollis Dejean | AF438033 |  |  |
|  | Rhadine sp. | AF438128 | AF437990 |  |
|  | Liagonum sp. | AF438090 |  |  |
|  | Olisthopus micans LeConte | AF438102 | AF437973 |  |
|  | Platynus hypolithos Say | AF438123 |  |  |
|  | Incagonum sp. | AF438079 |  |  |
|  | Atranus pubescens Dejean | AF438026 | AF437911 |  |
|  | Euplynes limbipennis Bates | AF438067 | AF437945 |  |
|  | Dicranoncus quadridens Motschulsky | AF438056 | AF37934 |  |
| Pseudomorphini | Pseudomorpha nr.angustata Horn | AF398714 | AF398622 | AF002782 |
|  | Sphallomorpha sp.1 | AF398679 | AF398636 | AF398717 |
|  | Sphallomorpha sp.2 | AF438133 | AF437995 |  |
|  | Adelotopus bolitus Castelnau | AF438010 |  |  |
| Harpalini | Harpaline | AF438072 | AF437950 |  |
| Pelmatellina | Pelmatellus sp. | AF398690 | AF398615 | AF398720 |
|  | Lecanomerus niger Darlington | AF438088 | AF437963 |  |
| Anisodactylina | Notiobia sp. | AF438101 | AF437972 |  |
| Stenolophina | Stenolophus (Egadroma) quinquepustulata Wiedmann | AF438137 | AF437999 |  |
|  | Bradycellus sp. | AF438032 | AF437916 |  |
| Harpalina | Discoderus cordicollis Horn | AF398652 | AF398588 | AF002776 |
|  | Harpalus caliginosus Fabricius | AF438073 | AF437951 |  |
| Dercylini | Dercylus sp. | AF438054 |  |  |
| Chlaeniini | Chlaenius ruficauda Chaudoir | AF398680 | AF398578 | AF002777 |
|  | Chlaenius (Lissauchenius) rufifemoralis bimaculatus Dejean | AF438038 | AF437921 |  |
|  | Chlaenius (Callistomimus) sp.2 | AF438039 | AF437922 |  |
| Panagaeini |  |  |  |  |
| Tefflina | Tefflus sp. | AF298703 | AF398630 |  |
| Panagaeina | Panagaeus sallei Chaudoir | AF398691 | AF398612 |  |
|  | Microschemus sp. | AF438096 | AF437968 |  |
|  | Craspedophorus rufipalpis LaFerte | AF438045 | AF437928 |  |
| Oodini | Anatrichis sp. | AF438015 | AF437903 |  |
|  | Adelopomorpha glabra | EU239505 |  |  |
|  | Stenocrepis elegans LeConte | AF398668 | AF398627 |  |
|  | Oodine sp.2 | AF438106 |  |  |
|  | Oodine sp.1 | AF438105 | AF437976 |  |
|  | Oodine sp.3 | AF438107 |  |  |
|  | Oodes amaroides Dejean | AF438104 | AF437975 |  |
| Licinini |  |  |  |  |
| Dicaelina | Dicaelus ambiguus LaFerte-Senectere | AF398655 | AF398586 |  |
|  | Diplocheila striatopunctata LeConte | AF438061 | AF437939 |  |
| Licinina | Badister neopulchellus Lindroth | AF438029 | AF437913 |  |
|  | Badister reflexus LeConte | AF438030 | AF437914 |  |
|  | Eutogeneius fuscus Solier | AF438070 | AF437948 |  |
|  | Licinus cassideus Fabricius | AF438090 |  |  |
| Dicrochilina | Dicrochile sp. | AF438058 | AF437936 |  |
| Lestignathina | Lacordairia sp. | AF438082 | AF437992 |  |
| Anthiini |  |  |  |  |
| Cypholobina | Cypholoba sp. | AF398695 | AF398584 |  |
| Anthiina | Anthia sp. | AF398696 | AF437906 |  |
| Physocrotaphini | Pogonoglossus sumatrensis Gestro | AF438125 |  |  |
| Helluonini |  |  |  |  |
| Helluonina | Gigadaema sp. | AF398662 |  |  |
|  | Aenigma iridis Newman | AF438012 |  |  |
|  | Dicranoglossus resplendens Castelnau | AF438055 |  |  |
| Omphrina | Helluomorphoides latitarsis Casey | AF398689 | AF398594 |  |
|  | Helluomorphoides oculeus Bates | AF438074 | AF37952 |  |
|  | Omphra sp. | AF398657 | AF398610 |  |
|  | Macrocheilus sp. | AF438092 | AF437965 |  |
| Orthogoniini | Orthogonius sp.1 | AF398709 | AF398611 | AF398719 |
|  | Orthogonius sp.2 | AF438109 |  |  |
|  | Orthogonius sp.3 | AF438110 |  |  |
| Galeritini |  |  |  |  |
| Planetina | Planetes ruficollis Nietner | AF438122 |  |  |
|  | Planetes sp. | AF438121 | AF437986 |  |
| Galeritina | Galerita lecontei lecontei Dejean | AF398686 | AF398590 | AF002780 |
|  | Galeritine | EU239508 |  |  |
|  | Trichognathus sp. | EU239509 |  |  |
|  | Ancystroglossus n. sp. | AF438018 |  |  |
| Dryptini | Drypta sp. | AF438064 |  |  |
|  | Desera australis Péringeuy | AF398659 | AF398585 |  |
| Zuphiini |  |  |  |  |
| Mischocephalina | Mischocephalus sp. | AF438098 |  |  |
| Patriziina | Thalpius nr. rufulus LeConte | AF398697 | AF398632 | AF002781 |
|  | Thalpius sp.1 | AF438145 | AF438004 |  |
|  | Pseudaptinus (Pseudaptinus) lecontei Dejean | AF438127 | AF37989 |  |
| Zuphiina | Zuphium sp.1 | AF398667 | AF398634 |  |
|  | Acrogenys sp. | EU239504 |  |  |
|  | Zuphium sp.2 | AF438147 | AF438006 |  |
| Hexagoniini | Hexagonia sp. | AF438075 | AF437953 |  |
|  | Dinopelma nr. immaculatum Andrews | AF438059 | AF437937 |  |
| Ctenodactylini | Ctenodactyla batesii Chaudoir | AF398688 | AF398582 |  |
|  | Leptotrachelus dorsalis Fabricius | AF398646 | AF398599 |  |
|  | Plagiotelum irinum Solier | AF438120 | AF437985 |  |
|  | Ctenodactyla sp.2 | AF438048 | AF437929 |  |
|  | Teukrus nr. cruciatus Bates | AF438144 | AF438003 |  |
| Lachnophorini | Calybe laetula LeConte | AF398705 | AF398576 | AF002772 |
|  | Aporesthus nr. anomalus Bates | AF438021 | AF437907 |  |
|  | Lachnophorus elegantulus Mannerheim | AF398650 | AF398597 |  |
|  | Anchonoderus sp. | AF438017 | AF437904 |  |
|  | Asklepia n. sp. | AF438024 |  |  |
|  | Euphorticus pubescens Dejean | AF438066 | AF437944 |  |
| Odocanthini | Colliuris pensylvanica Linné | AF398712 | AF398581 |  |
|  | Colliuris (Eucolliuris) sp.2 | AF438041 | AF437924 |  |
|  | Pentagonica roedingeri Liebke | AF398637 | AF398616 |  |
|  | Pentagonica nr. blanda Andrewes | AF438115 | AF437981 |  |
|  | Scopodes sp. | AF398656 | AF398626 |  |
|  | Actenonyx bembidiodes White | AF438009 | AF437898 |  |
|  | Lasiocera sp. | AF438083 | AF437958 |  |
|  | Dicraspeda brunnea Chaudoir | AF438057 | AF437935 |  |
|  | Clarencia sp. | AF438040 | AF437923 |  |
|  | Stenidia rugucollis Fairmaire | AF438134 | AF437996 |  |
|  | Ophionea ishii Habu | AF438108 |  |  |
| Calophaenini | Calophaena n. sp. | AF398666 | AF398575 |  |
|  | Calophaena nr. dupuisi Liebke | AF438036 | AF437919 |  |
| Perigonini | Perigona nigriceps Dejean | AF398665 | AF398617 |  |
|  | Perigona (Ripogena) bembidoides Alluaud | AF438129 |  |  |
|  | Diploharpus laevissimus Chaudoir | AF438062 | AF437940 |  |
| Lebiini | Demetrida dieffenbachia White | AF438053 | AF437933 |  |
|  | Brachyctis rugulosa Chaudoir | AF438031 | AF437915 |  |
|  | Celeanephes parallelus | EU239507 | EU239503 |  |
|  | Sinurus opacus Chaudoir | AF438131 | AF437993 |  |
| Pericalina | Stenognathus (Pristolomus) dentifer Chaudoir | AF438136 | AF437998 |  |
|  | Coptodera aerata Dejean | AF438042 | AF437925 |  |
|  | Pericalus quadrimaculata MacLeay | AF438117 | AF437982 |  |
|  | Inna breviformis Chaudoir | AF438080 | AF437957 |  |
|  | Catascopus sp. | AF438037 | AF437920 |  |
|  | Somotrichus elevatus Fabricius | AF438132 | AF437994 |  |
|  | Coptodera erotyloides Bates | AF438043 | AF437926 |  |
|  | Philophlaeus sp. | AF438118 | AF437983 |  |
|  | Serrimargo verrucifer Chaudoir | AF438130 | AF437991 |  |
|  | Pericaline | AF438116 |  |  |
|  | Menarus testaceus Jedlicka | AF438094 |  |  |
|  | Mormolyce hagenbachii Westwood | AF438099 | AF437970 |  |
|  | Pristacrus laticollis Gory&Castelnau | AF438126 | AF437988 |  |
|  | Thysanotus apicalis Alluaud | AF438146 | AF438005 |  |
|  | Coptoptera apicalis Péringuey | AF438044 | AF437927 |  |
|  | Stenotelus opacus Bouchard | AF438138 | AF438000 |  |
|  | Eurydera unicolor Klug | AF438069 | AF437947 |  |
|  | Antimerina elegans Alluaud | AF438020 |  |  |
| Agrina | Agra exarata group | AF438013 | AF437901 |  |
|  | Agra n. sp. truquii group | AF438014 | AF437902 |  |
| Apenina | Apenes hilariola Bates | AF398713 | AF398567 |  |
|  | Cymindoidea sp. | AF438052 | AF437932 |  |
| Calleidina | Calleida decora Fabricius | AF398663 | AF398574 |  |
|  | Mimodromius nigrotestaceus Solier | AF438097 | AF437969 |  |
|  | Onota angulicollis Reiche | AF438103 | AF437974 |  |
|  | Plochionus timidus Haldeman | AF438124 | AF437987 |  |
|  | Callidiola (Goniocallida) olsoufieffi Jeannel | AF438035 | AF437918 |  |
|  | Stenocallida augusticollis Boheman | AF438135 | AF437997 |  |
|  | Anomotarus (Nototarus) chaudoiri Sloane | AF438019 | AF437905 |  |
|  | Calleida (Philophuga) caerulea Casey | AF438034 | AF437917 |  |
| Cymindina | Cymindis (Pinacodera) puntigera LeConte | AF398651 | AF398583 | AF002773 |
|  | Cymindis (Cymindis) evanescens Casey | AF438050 |  |  |
|  | Cymindis (Taridius) stevensi Andrewes | AF438051 | AF437931 |  |
|  | Hystrichopus (Pseudomasoreus) reticulatus Ball&Hilchie | AF438076 | AF437954 |  |
|  | Hystrichopus (Hystrichopus) sp.1 | AF438077 | AF437955 |  |
|  | Hystrichopus sp.2 | AF438078 | AF437956 |  |
| Dromiina | Apristus sp. | AF438022 | AF437908 |  |
|  | Microlestes lucidious LeConte | AF438095 |  |  |
|  | Dromius piceus Dejean | AF438063 | AF437942 |  |
|  | Axinopalpus fusciceps LeConte | AF438028 |  |  |
|  | Axinopalpus biplagiatus Dejean | AF438027 | AF437912 |  |
|  | Syntomus sp. | AF438140 | AF437962 |  |
| Lebiina | Arsinoe nr. egregia Péringuey | AF438023 | AF437909 |  |
|  | Lebia viridis Dejean | AF398649 | AF398598 |  |
|  | Lebia pulchella Dejean | AF438084 | AF437959 |  |
|  | Lebia sp.1 | AF438085 | AF437960 |  |
|  | Lebia sp.2 | AF438086 | AF437961 |  |
|  | Lebia sp.3 | AF438087 |  |  |
|  | Lachnoderma hirsutum Bates | AF438081 |  |  |
|  | Physodera n. sp. nr. bifenestrata Heller | AF438119 | AF437984 |  |
|  | Endynomena sp. | AF438065 | AF437943 |  |
|  | Aspasiola n.sp. | AF438025 | AF437910 |  |
|  | Lia quadrinotata Chevrolat | AF438089 | AF437964 |  |
|  | Hyboptera angulicollis Chaudoir | AF398664 | AF398595 |  |
| Metallicina | Euproctinus pallidus Shpeley | AF438068 | AF437946 |  |
|  | Parena picea MacLeay | AF438113 | AF437979 |  |
| Demetriina | Cylindrocranius sp. | AF438049 | AF437930 |  |
|  | Peliocypas sp. | AF438114 | AF437980 |  |
| Graphipterini | Graphipterus cordiger Dejean | AF398711 | AF398598 |  |
|  | Graphipterus limbatus Castelnau | AF438071 | AF437949 |  |
| Cyclosomini | Masoreus wetterhalli axillaris Gyllenhal | AF438093 | AF437967 |  |
|  | Tetragonoderus insignicollis Chaudoir | AF438143 | AF437966 |  |
|  | Tetragonoderus chalceus Chaudoir | AF438142 | AF437941 |  |
|  | Tetragonoderus latipennis LeConte | AF398653 | AF398631 | AF012471 |
|  | Aephnidius sp. | EU239506 |  |  |
|  | Anaulacus (Macrancanthus) n. sp. | AF438016 | AF439790 |  |
|  | Sarothrocrepis sp. | AF398670 | AF398624 |  |
